# Intake of Fibre-Associated Foods and Texture Preferences in Relation to Weight Status Among 9–12 Years Old Children in 6 European Countries

**DOI:** 10.3389/fnut.2021.633807

**Published:** 2021-02-18

**Authors:** Marlies Hörmann-Wallner, Raphaela Krause, Begoña Alfaro, Hannah Jilani, Monica Laureati, Valérie L. Almli, Mari Sandell, Pernilla Sandvik, Gertrude G. Zeinstra, Lisa Methven

**Affiliations:** ^1^Institute of Dietetics and Nutrition, University of Applied Sciences FH JOANNEUM, Graz, Austria; ^2^Food Research Division, Expert Technology Centre in Marine and Food Innovation (AZTI), Derio Bizkaia, Spain; ^3^Institute for Public Health and Nursing Research– Leibniz Institute for Prevention Research and Epidemiology, University of Bremen and Institute for Preventions Research and Epidemiology– Instituts für Public Health und Pflegeforschung, Bremen, Germany; ^4^Department of Food, Environmental and Nutritional Sciences, University of Milan, Milan, Italy; ^5^Department of Innovation, Consumer and Sensory Sciences, Nofima, Aas, Norway; ^6^Functional Foods Forum, University of Turku, Turku, Finland; ^7^Department of Food and Nutrition, University of Helsinki, Helsinki, Finland; ^8^Department of Food Studies, Nutrition and Dietetics, Uppsala University, Uppsala, Sweden; ^9^Food, Health & Consumer Research Group, Wageningen Food & Biobased Research, Wageningen, Netherlands; ^10^Department of Food and Nutritional Sciences, University of Reading, Reading, United Kingdom

**Keywords:** texture preferences, plant foods, high/low fibre foods, overweight, BMI-for-age percentiles

## Abstract

Plant foods, rich in fibre, can offer textures that children find difficult to orally manipulate, resulting in low preferences but are important for a healthy diet and prevention of overweight in children. Our aim was to investigate preferences for food texture, intake of fibre-associated foods and the relation to BMI. Three hundred thirty European children (9–12 years, 54% female) indicated their texture preferences using the Child-Food-Texture-Preference- Questionnaire (CFTPQ), and their parents responded on fibre-associated food consumption and anthropometric information. BMI was significantly lower for children with higher intake of wholegrain alternatives of common foods; in addition to being significantly influenced by country and the wearing of a dental brace. Overall BMI-for-age-percentiles (BMI_pct) were negatively associated with the consumption of wholegrain cereals, white pasta and wholemeal products and positively associated with the intake of legumes and white biscuits. In males, BMI_pct were negatively associated with wholegrain products and dried fruits, and in females, positively with legume consumption. A few country-related associations were found for BMI_pct and wholegrain biscuits, seeds and nuts and refined products. No overall correlation was found between BMI_pct and the texture preference of soft/hard foods by CFTPQ, except in Austria. We conclude that this study revealed evidence of a connection between fibre-associated foods and children‘s BMI at a cross-cultural level and that sex is an important determinant of fibre-associated food intake and the development of overweight in childhood.

## Introduction

Nowadays childhood overweight/obesity is one of the most significant health problems worldwide (1). The highest levels of children with overweight and obesity have been reported in Southern Europe (Greece, Italy, Spain), whereas the ratio tends to be lower in Western and Northern Europe (2). Overweight and obesity existing already in childhood and adolescence can lead to serious chronic diseases (3, 4) and when persisting into adulthood, cardiovascular disease, diabetes and certain cancers are serious consequences (5–7). Therefore, the evaluation and classification of weight status in children is important for health assessment and- monitoring the prevention of diseases (8).

Observational studies have consistently found a positive correlation between the consumption of plant foods and improved long-term health outcomes in children (9, 10) emphasising that dietary fibre-rich plant foods, such as grains, fruits, vegetables, potatoes and legumes have positive health effects. The main effects are weight loss (10) and reduced long term risk of metabolic syndrome (11). Studies conducted in Europe, though carried out with adults, confirmed that intake of fibre-rich wholegrain pasta compared to refined pasta increased satiety and reduced hunger without changing the energy intake at subsequent meals (12, 13).

However, the intake of fruits and vegetables in European children is low (14), suggesting that only 6–24% reach the WHO recommendations, where females show a higher consumption than males (15–18). Moreover, research confirms that children with overweight/obesity consume less fruits and vegetables than children with normal weight (19). A low consumption of fruits and vegetables is not only associated with a higher risk of becoming overweight (19, 20), but also with the development of chronic diseases (21, 22). It was found that many children, instead of a diet rich in fruits and vegetables, choose rather sugar- and fat-rich snacks (23–25), which could lead to a higher energy intake (26) and consequently to overweight/obesity (27–29). However, no difference in the number of daily snacks between overweight and children with normal weight was found (23).

Eating behaviours in childhood are also often affected by picky eating (30), whereas these factors may have an influence on the development of overweight/obesity. Picky eating is very common in childhood, because children often have an avoidance of certain foods (31) and therefore show a low variety of foods in their diet.

The reasons for picky eating may be due to infancy, because food texture and the acceptance of food were related to the development of chewing (32). It has been shown that children who have early experiences with texture in their life tend to show a broader acceptance of those textures later. Demonteil et al. found that children were able to accept most textures at the age of 12 month because their chewing behaviour was established, however this was not always reflected by parental feeding practices (33). Lukasewycz and Mennella showed in their study that adults prefer harder foods and those containing more particles and children's preferences are developing with increasing age so that they become more adult-like later on (32). Szczesniak found out that children prefer food that can be easily manipulated in mouth (34). Moreover, a study from Werthmann et al. showed that children's consumption of e.g., a yoghurt decreased when the texture was modified by adding pieces to it (31). In vegetables tactile properties play a particularly important role in the rejection or acceptance (35). Therefore, the consumption of vegetables in young people is strongly related to the sensory properties of the respective vegetable variety (36–39). The structure of plants is based upon fibre, therefore, the texture of fibre-rich foods have more intensive tactile properties which may lead some children to reject such foods (31, 35). Studies show that children often reject foods with slimy, grainy or hard textures (36–39).

Little is yet known about a possible correlation between food texture preferences and overweight in children. In one previous European study a correlation was found between the preference for foods with a soft texture and food neophobia (40), which is in turn linked to the increase in body weight in adults (41).

The present study aimed to explore the relationship between weight status of European children, the consumption frequency of foods with high and low fibre content and texture preferences (soft/hard). We hypothesised that; (1) A higher intake of high fibre foods would be correlated with a lower BMI in children, potentially due to increasing satiety and reducing the intake of energy-dense foods, and (2) A higher preference for soft textures and therefore a lower consumption of plant-based high fibre foods would be connected with a higher BMI in children.

## Materials and Methods

### Study Design and Participants

**Figure 1:**
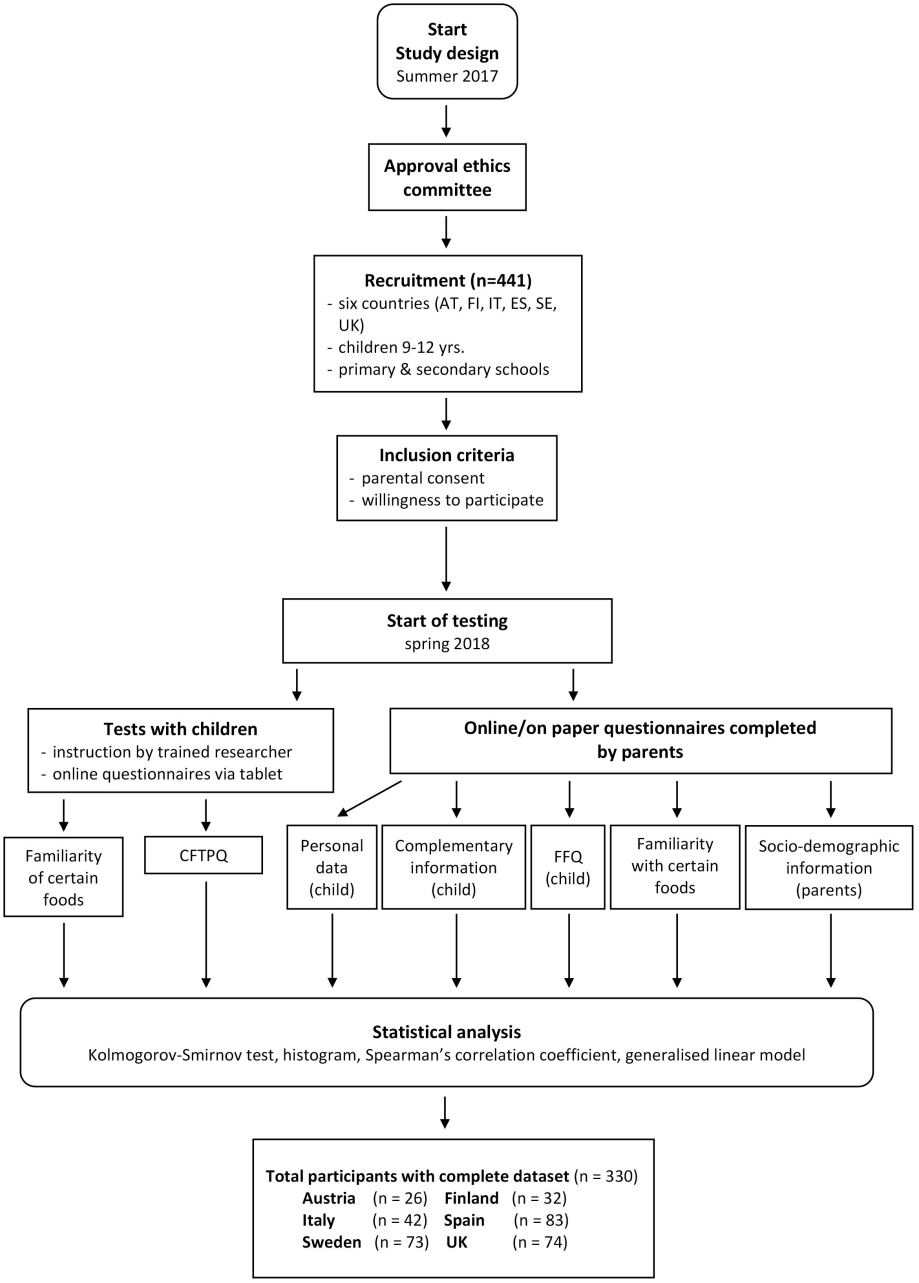
Flow chart of the study.

A total response of 330 children (9–12 years, 151 males, 179 females) from six European countries (Austria, Finland, Italy, Spain, Sweden and UK) and their parents was reached in this study (see Figure 1). The number of participants from each country is summarised in Table 1. The study protocol was approved by the relevant research ethics committee of each country, and written consent was obtained from the parents according to the declaration of Helsinki (Austria: No. 30-200 ex. 17/18, Finland: No. 12/2018, Italy: No. 49/17, Spain: No. PI2017180, Sweden: No 2017/549, UK: No. UREC 18/15). Inclusion criteria were the parental consent and the willingness to participate within this study. In detail, parents were informed about the procedures and were asked to sign an informed consent and therefore, children without a signed parental informed consent were excluded from the study. Children received an information sheet and gave their verbal consent to participate, none of the children declined to participate to the study. Recruitment took place in both primary and secondary schools, as children with the age between 9 and 12 years are sufficiently evolved to understand and complete the selected sensory tests (see below) (42). The study design included tests with the children which were carried out directly at school during class, as one visit per class. The parents completed questionnaires relating to their children at home via on-line links, or on-paper, as preferred by the parent. These questionnaires included personal data of their children (e.g., age, sex, height, weight), eating habits of their children (food frequency questionnaire, FFQ, mainly on fibre-rich and fibre-low foods), complementary information on their children (e.g., dental status), their familiarity with certain food items, and gave information on socio-demographic information (e.g., economic situation, education). Questionnaires and procedures for both children and parents were translated in English, reviewed by a native English speaker, and then translated in every language by two independent native speakers. The two translated versions were compared to identify discrepancies and reach consensus for an updated version. To improve comparability of the data collected in different cultures (43), procedures, experimental design and instructions to children and parents were the same in all countries and all tests were carried out within a 3-month period in the spring of 2018. After a team of trained researchers gave instructions, children worked on their own answering the questionnaires online via tablets. Children were tested by class or in smaller groups (4–5 children) on their own and were not influenced by their classmates. Tablets/computers were used by the children to complete the online questionnaire on familiarity with certain food items and the child food texture preference questionnaire (CFTPQ).

**Table 1:** Distribution of weight status.

		**Country**						**Sex**	
BMI classification^*^	**Total**	**Austria**	**Finland**	**Italy**	**Spain**	**Sweden**	**UK**	**Males**	**Females**
	***n* (%)**	***n* (%)**	***n* (%)**	***n* (%)**	***n* (%)**	***n* (%)**	***n* (%)**	***n* (%)**	***n* (%)**
Underweight <5 pct	23 (7.0)	3 (11.5)	3 (9.4)	4 (9.5)	6 (7.2)	4 (5.5)	3 (4.1)	9 (6.0)	14 (7.8)
Normal weight 5–84.9 pct	252 (76.4)	18 (69.2)	23 (71.9)	35 (83.3)	60 (72.3)	58 (79.5)	58 (78.4)	113 (74.8)	139 (77.7)
Overweight ≥ 85 pct	55 (16.7)	5 (19.2)	6 (18.8)	3 (7.1)	17 (20.5)	11 (15.1)	13 (17.6)	29 (19.2)	26 (14.5)
*Overweight 85–94.9 pct*	*40 (12.1)*	*4 (15.4)*	*5 (15.6)*	*2 (4.8)*	*12 (14.5)*	*9 (12.3)*	*8 (10.8)*	*23 (15.2)*	*17 (9.5)*
*Obese ≥ 95 pct*	*15 (4.6)*	*1 (3.8)*	*1 (3.1)*	*1 (2.4)*	*5 (6.0)*	*2 (2.7)*	*5 (6.8)*	*6 (4.0)*	*9 (5.0)*
Total	330 (100)	26 (100)	32 (100)	42 (100)	83 (100)	73 (100)	74 (100)	151 (100)	179 (100)

* *Distribution of BMI categories according to CDC criteria in participating countries, pct=BMI-for-age-percentiles*.

The data presented in this paper were collected as part of a wider study that developed and validated the child food preference questionnaire (CFTPQ), and further details of the overall study design are reported in Laureati et al. (40).

### Anthropometric Measurements

In all six countries, the weight and the height of the children were self-reported by the parents within an online questionnaire. In addition, in Austria and the United Kingdom, the weight in kg (nearest to 0.1 kg) of the children was obtained with a portable scale (SECA). To determine children's height in cm (nearest to 0.1 cm) a portable stadiometer (SECA) in Austria and a tape measure mounted on the wall in the United Kingdom were used. The BMI was calculated by dividing the body weight in kg by the square of the height in m. High correlation coefficients of self-reported vs. measured BMI-data were obtained in UK (*r* = 0.82, *p* < 0.01) and Austria (*r* = 0.94, *p* < 0.01). This self-report validity could only be checked for UK and Austria, as there were no measured data in the other four countries. In addition to reporting numerical BMI data, BMI-for-age percentiles (BMI_pct) data are also reported in this paper. This accounts for the fact that children are individually and rapidly growing, hence the classification of weight status during childhood is complex (8). The Centers for Disease Control and Prevention (CDC), the International Obesity Task Force (IOTF) and the World Health Organisation (WHO) have developed suitable methods for BMI classification in children (44–46). CDC BMI_pct, suitable for comparing the BMI-data from different countries were used and for each participant the percentile was calculated. Furthermore, the BMI of participants was grouped into underweight (<5th percentile), normal weight (5th–84.9th percentile) or overweight (85th−94.9th percentile)/obesity (≥95th percentile) according to age- and sex-specific BMI percentiles cut-offs (46).

### Child Food Texture Preference Questionnaire

The detailed procedure for data collection and calculation of the CFTPQ index was previously described elsewhere (40). In brief, a questionnaire with pictures of 17 pairs of soft/smooth and hard/particulate food were presented to the child, e.g., yoghurt with pieces/yoghurt without pieces, apple/apple sauce, orange/orange juice, or toasted bread/soft bread. Of each pair, the child chose the preferred one. The children also reported their familiarity with each item. When both food pictures of a pair were also reported familiar by the child, this pair was considered as valid an included within the analysis. Children with <8 valid pairs were excluded from analysis resulting in 309 datasets from 330 children. For each time choosing the soft/smooth version a value of 1, or for choosing the hard/particulate version a value of 2 was given. Individual CFTPQ indices were calculated. The score ranged from 0 to 100 with higher scores representing a preference for the hard/particulate food items.

### Food Frequency Questionnaire for High/Low Fibre Foods

A 17-item questionnaire (40) of the frequency consumption of 12 fibre-rich (e.g., wholegrain products) and 5 low-fibre (e.g., refined “white” products) foods was completed by the parents for their children. Unreturned and incomplete questionnaires could not be included from the analysis (*n* = 111), resulting in *n* = 330 valid answers. Data were collected using a 6-point-category scale with answering options, less than once a month or never, 1–3 times per month, 1–3 times a week, 4–6 times a week, once a day, multiple times per day; in addition to category, I don't know. Parents were asked to recall their child‘s intake in the aforementioned categories over the previous 4—week period, and images were provided with the name of each food item. For each food item, the daily frequency equivalents (DFE) were calculated, so that the daily consumption quantity of all foods can be compared. DFE of 0= less than once a month or never, DFE of 0.07 = 1–3 times a month, DFE of 0.28 = 1–3 times a week, DFE of 0.71 = 4–6 times a week, DFE of 1 = once a day, DFE of 2.5 = multiple times a day, as published elsewhere (40).

Furthermore, the following scores were calculated from the FFQ Items:

**Total consumption of high fibre foods (12 items, DFE):** DFE sum of high fibre foods

∑ [wholegrain versions of bread, porridge, cereals, biscuits, rice and pasta; fresh fruits, dried fruits, nuts/seeds, vegetables, potatoes, legumes]

**Total consumption of wholemeal foods (5 items, DFE):** DFE sum of wholegrain foods

∑ [wholegrain versions of bread, cereals, biscuits, rice, pasta]

**Total consumption of refined foods (5 items, DFE):** DFE sum of refined foods

∑ [refined versions of bread, cereals, biscuits, rice, pasta]

**Wholegrain (%):**

$$\begin{array}{l} \frac{wholemeal\;alternatives\;}{refined\;alternatives}\times 100 \end{array}$$

### Statistical Analysis

Distribution of data was tested with one sample Kolmogorov-Smirnov tests and histograms. Outlier analysis confirmed there were no outliers in BMI nor CFTPQ variables. As expected there were outliers in reported FFQ data, as the majority of children having low wholegrain consumption. However, this reflects current dietary habits and, therefore, as consumption of higher levels of wholegrain foods is of interest in this study the outliers were not removed in the primary analysis. Where significant effects relating consumption frequency to BMI were found secondary analysis was carried out excluding outliers and subsequently reported in the limitations section.

The association between BMI_pct and the consumption frequencies of specific food items and the CFTPQ index were tested with Spearman‘s correlation coefficient (Table 2). To assess the related effect of independent factors and covariates, the generalised linear model (GsLM, BMI_pct as dependent variable) was included in the analyses. As the BMI-for-age percentiles are important in the consideration of children‘s BMI and considering most of data were not normally distributed, including any transformation of BMI_pct, the generalised linear model analysis using the Tweedie model was carried out. Included (significant) factors and covariates were the country of participation, currently wearing a dental brace and total consumption of wholemeal foods (section Relationship between BMI-for-age percentiles and all other factors) whereas sex, age of introducing solids, whether parents went to university, economic situation, CFTPQ, total consumption of refined alternatives, high fibre foods and wholegrain (%) were excluded. Subsequently, single food items that significantly correlated with BMI_pct were tested within (wholegrain cereals, white biscuits, white pasta, and legumes). Four final models with one or more factor/covariate were built (models 1–4).

**Table 2:** Spearman‘s correlation coefficient of the BMI-for-age percentiles (CDC) with texture preference and eating frequency.

	**Total**	**Austria**	**Finland**	**Italy**	**Spain**	**Sweden**	**UK**	**Males**	**Females**
	**(*n* = 309)**	**(*n* = 22)**	**(*n* = 32)**	**(*n* = 40)**	**(*n* = 74)**	**(*n* = 69)**	**(*n* = 72)**	**(*n* = 143)**	**(*n* = 166)**
CFTPQ-Index (by children)	0.056	0.441^*^	−0.104	0.029	0.163	−0.022	0.039	0.144(^*^)	−0.013
Food items and Indices (by adults)	Total (*n* = 330)	Austria (*n* = 26)	Finland (*n* = 25)	Italy (*n* = 42)	Spain (*n* = 83)	Sweden (*n* = 73)	UK (*n* = 74)	Males (*n* = 150)	Females (*n* = 173)
White bread (DFE)	−0.001	−0.101	−0.305	−0.091	0.183 (^*^)	0.081	−0.041	0.077	−0.062
Wholegrain bread (DFE)	−0.054	0.024	−0.050	0.103	−0.035	−0.045	−0.138	−0.158(^*^)	0.061
Wholegrain porridge (DFE)	0.010	0.231	−0.049	−0.125	0.045	0.064	0.031	−0.115	0.135
Cornflakes (DFE)	−0.041	−0.265	−0.118	0.010	0.029	−0.106	−0.087	−0.065	−0.028
Wholegrain cereals (DFE)	−0.127^*^	0.130	−0.181	−0.289(^*^)	−0.099	−0.160	−0.176	−0.225^**^	−0.031
Biscuits white (DFE)	0.119^*^	−0.258	−0.172	0.151	0.099	0.084	0.088	0.123	0.116
Wholegrain biscuits (DFE)	−0.001	−0.007	−0.431^*^	0.028	0.028	0.252^*^	0.107	−0.065	0.069
Fresh fruits (DFE)	−0.032	−0.048	0.010	−0.251	−0.037	0.003	−0.035	−0.036	−0.018
Dried fruits (DFE)	−0.089	0.052	−0.292	−0.009	−0.105	−0.062	−0.154	−0.229^**^	0.048
Total fruits (fresh and dried) (DFE)	−0.032	−0.050	−0.129	−0.261	−0.073	−0.029	−0.077	−0.100	0.003
Seeds and nuts (DFE)	−0.002	−0.182	−0.201	0.177	−0.226^*^	0.276^*^	−0.025	−0.069	0.065
Vegetables (DFE)	−0.027	−0.099	0.022	−0.206	0.121	0.055	−0.045	−0.041	0.015
Potatoes (DFE)	−0.020	0.027	0.077	0.055	−0.078	0.019	−0.117	−0.060	−0.007
Legumes (DFE)	0.145^**^	0.036	0.235	−0.066	0.203(^*^)	0.066	0.210(^*^)	0.043	0.227^**^
Rice white (DFE)	−0.016	−0.169	−0.370(^*^)	−0.005	0.110	−0.135	0.074	−0.042	0.013
Wholegrain rice (DFE)	−0.042	0.149	−0.101	0.068	−0.079	0.080	−0.182	−0.106	0.034
Pasta white (DFE)	−0.122^*^	−0.118	−0.180	−0.249	−0.030	−0.089	−0.046	−0.106	−0.145(^*^)
Wholegrain pasta (DFE)	−0.028	−0.005	−0.224	0.089	−0.028	0.110	0.059	−0.096	0.068
Vegetables and fruits (DFE)	−0.049	−0.080	−0.007	−0.246	−0.004	0.018	−0.068	−0.063	−0.022
TC of high fibre foods (DFE)^†^	−0.048	0.072	−0.089	−0.240	−0.077	0.076	−0.034	−0.111	0.048
TC of wholemeal foods (DFE)^‡^	−0.113^*^	0.160	−0.231	−0.075	−0.146	0.006	−0.126	−0.229^**^	0.022
TC of refined foods (DFE)^§^	0.014	−0.164	−0.321	−0.073	0.239^*^	−0.090	−0.025	0.041	−0.018
Wholegrain (%)^||^	−0.067	0.305	0.107	−0.041	−0.188(^*^)	0.089	−0.084	−0.201^*^	0.086

Comparison of texture preference (CFTPQ, child food texture preference questionnaire) and correlation of frequency consumption (daily frequency equivalents, DFE) of certain fibrerich/poor foods and Scores of fibrerich/poor foods reported by adults, TC, Total consumption; (DFE), daily frequency equivalents, (*) only borderline significant, but maybe worthy of an additional investigation p ≤ 0.1,

* *p ≤ 0.05*,

** *p ≤ 0.01*.

† *Total consumption of high fibre foods (12 items, DFE): Sum of daily consumption of high fibre foods (wholegrain: bread, porridge, cereals, biscuits, fresh fruit, dried fruit, seeds, vegetables, potatoes, legumes, rice, pasta)*.

‡ *Total consumption of wholemeal foods (5 items, DFE): Sum of daily consumption of whole meal foods (bread, cereals, biscuits, rice, pasta)*.

§ *Total consumption refined foods (5 items, DFE): Sum of refined foods (bread, cereals, biscuits, rice, pasta)*.

|| *Wholegrain (%): (whole-meal alternatives/refined alternatives) × 100*.

Effects showing a *p* ≤ 0.05 were considered significant, while *p* ≤ 0.10, although not significant but at a borderline level (47), were marked and reported as observed differences that may be worthy of additional investigation. The analysis was performed with SPSS V24 Software (IBM Analytics, USA).

## Results

### Weight Status Across Country and Sex

Out of the 330 participating children with completed parental questionnaire, 76.4% had normal weight, 7.0% had underweight, 16.7% had overweight, whereof 4.6% had obesity (Table 1).

Taking children with overweight and obesity together, the highest proportions were reported for Spain (20.5%), followed by Austria (19.2%), Finland (18.8%), UK (17.6%). The countries with the lowest proportion of children with overweight and obesity were Sweden (15.1%) and Italy (7.1%). Partly, due to low subject numbers in many groups the differences between countries could not revealed significant.

Almost 6.0% of males and 7.8% of females had underweight, whereas 19.2% of males had overweight or obesity compared to 14.5% of females. The differences between males and females were only borderline significant (difference between males and females with overweight was *p* = 0.087).

### Relationships Between BMI_pct and Texture Preference

Preference for softer (smooth) or harder (particulate) textures concluded from the CFTPQ index was not significantly correlated with the BMI_pct (*r* = 0.056, *p* = 0.998, *n* = 309). Austria was the only country showing a significant positive correlation between preference for harder/particulate textures (higher CFTPQ) and BMI_pct (*r* = 0.441, *p* = 0.040, *n* = 22); the positive correlation in males between texture preferences (CFTPQ) and BMI_pct was borderline significant (*r* = 0.144, *p* = 0.086, *n* = 143) (Table 2).

### Associations of BMI_pct and Consumption of High/Low Fibre Foods

Parental responses on the 17-item food frequency questionnaire were completed and returned for 330 of the 441 participating children (Table 2). BMI_pct across the overall group correlated negatively with the reported consumption of wholegrain cereals (*r* = −0.127, *p* = 0.021), white pasta (*r* = −0.122, *p* = 0.027) and wholemeal products (*r* = −0.113, *p* = 0.04). Furthermore, BMI_pct correlated positively with the consumption frequency of white biscuits (*r* = 0.119, *p* = 0.03) and legumes (*r* = 0.145, *p* = 0.008). In summary, children with higher weight consumed less wholegrain cereals, white pasta and wholemeal products, whereas they consumed more white biscuits and legumes.

### Country-Related Differences

There were a number of significant, mostly weak correlations of consumption frequency and BMI_pct that were found in the data from specific countries (Table 2).

In **Austria** (*n* = 26), there was no specific correlation with the BMI_pct and any food item.

Regarding **Finland** (*n* = 25), there was a negative correlation with whole grain biscuits (*r* = −0.431, *p* = 0.032) and a negative correlation for white rice with borderline significance (*r* = −0.370, *p* = 0.069).

For **Italy** (*n* = 42) it was revealed that the consumption of wholegrain cereals was negatively associated with BMI_pct, but not significantly (*r* = −0.289, *p* = 0.064).

In **Spain** (*n* = 77), there was a significant positive correlation between BMI_pct and the consumption of refined products (*r* = 0.239, *p* = 0.029), although this only reached borderline significance for white bread (*r* = 0.183, *p* = 0.097) and legume consumption (*r* = 0.203, *p* = 0.066). Furthermore, a significant negative correlation was found between BMI_pct and consumption of seeds and nuts (*r* =-0.226, *p* = 0.040), although the negative correlation with wholegrain (%) was only borderline significant (*r* = −0.188, *p* = 0.088).

Within the data of **Sweden** (*n* = 72), two positive correlations between BMI_pct and the consumption of whole grain biscuits (*r* = 0.252, *p* = 0.031) and seeds and nuts (*r* = 0.276, *p* = 0.018) was found.

Finally, in the **UK** (*n* = 74) a positive correlation but only borderline significant was found for the consumption of legumes and BMI_pct (*r* = 0.210, *p* = 0.073).Sex-related differences

Males with higher BMI_pcts consumed less **dried fruits** (*r* = −0.229, *p* = 0.005). Furthermore, the consumption of wholegrain products decreased with increasing BMI_pct (**wholemeal products** (*r* = −0.229, *p* = 0.005), wholegrain cereals (*r* = −0.225, *p* = 0.006), and only borderline significantly wholegrain bread (*r* = −0.158, *p* = 0.054) and wholegrains (%) (*r* = −0.201, *p* = 0.014).

In females, the BMI_pct positively correlated with the consumption of legumes (*r* = 0.227, *p* = 0.003) and negatively but only borderline significantly with the consumption of **white pasta** (*r* = −0.145, *p* = 0.069). No further sex-related correlations were revealed.

### Relationship Between BMI_pct and All Other Factors

Additional analysis was carried out to investigate relationships between BMI_pct, socio-demographic, texture preference, and food intake. GsLM found that country had no significant effect on BMI_pct (*p* = 0.095, model 1), however, both wearing braces (*p* = 0.045, *B* = −10.75) and total consumption of wholemeal foods were significantly related to lower BMI_pct (*p* = 0.037, *B* = −4.56, model 2). The median BMI_pct for those wearing a dental brace was 37 compared to 55 for those without. There was no overall significant effect of texture preference (CFTPQ) on BMI_pct. Separate models were performed to check the effects of reported consumption of individual foods, each model considering groups of food items which did not correlate with one another. In model 3 a significant relationship was found between BMI_pct and wholegrain cereals (*p* = 0.034, *B* = −10.17), white pasta (*p* = 0.020, *B* = −8.698) but not for white biscuits (*p* = 0.347) and in model 4 between BMI_pct and legumes (*p* = 0.041, *B* = 16.05) and wholegrain cereals (*p* = 0.034, *B* = −9.93).

Once the outlying data were removed, secondary analysis (regression and GsLM) found no significant relationship between wholegrain consumption and BMI_pct.

## Discussion

This study aimed to explore the relationship between the weight status of 9–12 years old children across Europe, their consumption of fibre rich foods and their preference for soft or hard food textures. The main findings are:

◦ The **proportion of** overall children with overweight or obesity was 16.7% and with underweight 7.0%.
◦ BMI_pct had significant negative associations with **the overall consumption frequency** of wholegrain cereals, white pasta and wholemeal products, and was positively associated with consumption frequency of legumes and white biscuits.
◦ **Country-related associations** were found for BMI_pct and wholegrain biscuits (negative, Finland; positive, Sweden), seeds and nuts (negative, Spain; positive Sweden) and refined products (positive, Spain).
◦ BMI_pct had a significantly negative association with frequency of consuming wholemeal (-products) and dried fruits, but only **in males**.
◦ The BMI_pct was significantly higher with a higher consumption of legumes, but only **in females**.
◦ No differences in texture preference (CFTPQ) for hard/particulate or soft/smooth food pairs according to BMI_pct were found, except a significant positive correlation for Austria.

### Weight Status and Covariates

The prevalence of children with overweight (including obesity) was found from highest to lowest in Spain (20.5%), Finland (18.8%), Austria (19.2%), the UK (17.6%), Sweden (15.1%), and by far lowest in Italy (7.1%). With the exception of Spain, our study found lower counts of children with overweight compared to literature (Spain: 19.2%, Finland: 23%, Austria: 25.2%, UK: 28%, Sweden: 17.7%, Italy: 20.8%) (48–51).

We assume that the lower reported distribution of overweight in these countries in our study might be explained by underreporting (52, 53) and/or the fact that the data were recorded in defined regions and are not representative for the whole country. For example, previous data has shown that inhabitants of Northern Italy have lower weight compared to people in the South (54, 55), and in line with this, children in our study were recruited in Northern Italy (Milano, Lombardy region). In England, the proportion of children with obesity has been found to be higher in areas of deprivation, significantly so for females (51). In the UK, part of the children in our study were recruited from urban areas in the South East which is not a region of high deprivation in England (56).

In Austria and the UK, measured and reported parental data of the children‘s height and weight (calculated BMI) had a very high agreement (respectively, 0.94 and 0.82, both *p* < 0.001). Furthermore, the use of different criteria in classification of weight status might limit the comparison between study results. In children, BMI for age and sex specific cut-off points are used according to population specific data e.g., WHO (45), CDC (46), or IOTF (44, 57). In our case, the CDC BMI-for-age percentiles were used. This classification is slightly different compared to the WHO criteria (57). There are high contrasts across different studies, as in an Italian sample only 2.7% children with underweight and 20.9% children with overweight were reported using WHO criteria (58).

There is less information on the proportion of children with underweight from studies in Europe compared to studies revealing overweight. Our study found rates of >7% in 4 out of 6 countries, in Italy (9.5%), Finland (9.4%), Austria (11.5%), and Spain (7.2%) still considering that these were self-reported data. In Spain, for 6–14 years old children, a mean prevalence of 10.2% for underweight was reported (59) using IOTF criteria, which is comparable to the present results.

Interestingly, we did not observe a tendency towards a lower BMI in southern compared to northern countries which was also noted in two recent epidemiological studies (2, 60). One might speculate, that diet styles might be changing, from the traditional southern Mediterranean diet, that is considered rich in fruits, vegetables and olive oil (61), towards more snacking and consumption of highly processed foods (62). This issue was addressed in recent studies found that a large part of the population of children and adolescents in the Southern countries have poor adherence to their traditional diet (63, 64). On the other hand, non-Mediterranean populations have been the major benefactors, since during the same period they have adopted a Mediterranean dietary pattern (65).

Another interesting result was that wearing a dental brace was accounting for a lower BMI (GsLM models; section 3.6) and this issue was already addressed elsewhere (66, 67). It is assumed that manipulating food is more difficult (68) and total food consumption might be lower (67) compared to children without wearing a dental brace. One previous study has reported mean body weight to significantly decrease during orthodontic treatment (from pre-treatment to 1 month into treatment), predominantly due to discomfort, however the study was of a limited size (*n* = 30) and in adults rather than children (66) Furthermore, a previous study found that increasing leptin-levels were reported in adults with orthodontic appliances, which might also lead to lower appetite and therefore to reduced food intake (69) and might contribute to a reduced BMI. In this context previous orthodontic research has found that adolescents with obesity had an increased initial tooth displacement and a higher rate of tooth movement compared to a normal-weight group, and it has been proposed that this may affect the treatment period (70–72), and therefore it is perhaps unsurprising that we found a difference in BMI related to wearing a dental brace.

### Weight Status & Texture Preference

We did not find any association between children's weight status and the preference for soft/smooth or harder/particulate structure of foods reflected by the CFTPQ index, either across the study population as a whole or country wise, except for Austria. In Austria the correlation was significant and quite high (*r* = 0.441), however the number of children was low (*n* = 22) and should be interpreted carefully. So to speculate for Austria it is indicated that with increasing weight children do like the particulate/harder foods e.g., chocolate bar, crunchy cornflakes or whole carrots. This is an unexpected result as food requiring more oral processing (e.g., hard, crunchy, thick food) has previously been associated with lower eating rate, a consequent increase of satiety and reduced energy intake (73). One study of eating rate in children (4–5 years) found that those who ate faster had higher energy intake, and this was associated with increased BMI z-score and adiposity (74). It should be underlined that texture preferences were investigated with a picture-based questionnaire (CFTPQ) with items that were not necessarily representative of fibre-rich foods, and thus may only partially have captured the link between fibre consumption and texture-driven preferences. More research is needed to better understand this complex and interrelated association.

### Weight Status & Consumption of High/Low Fibre Foods

Fibre consumption and consumption of fibre-rich foods in European countries does not meet current recommended daily intake guidelines (75–80), thus we anticipated that a consumption of high fibre foods would be negatively associated with BMI as indicated in literature (81, 82). In this study we found a weak, but significant association of increasing BMI_pct with a decreasing consumption frequency of wholegrain cereals and wholemeal products (bread, cereals, biscuits, pasta, rice). These results are mainly driven by Italy (cereals), Finland, Spain and the UK (wholemeal products) and by males (Table 2). Furthermore, both wholegrain products and wholegrain cereals were significant predictors of BMI percentiles, across the respective regression and GsLM models (section Relationship between BMI-for-age percentiles and all other factors). Koo et al. previously stated the importance of increasing wholegrain consumption on the management of childhood obesity (83), although studies show diverse results. In adults, within an intervention trial (*n* = 316) it was revealed that wholegrain consumption did not reduce body weight or fat (%) (10, 84). However, systematic reviews have concluded that the intake of high fibre cereals can lead to modest weight reduction in adults (10, 85). This was further confirmed by another survey (*n* = 716) on fibre intake in adolescents in schools. In one study it was revealed that fibre intake below the recommendations is associated with a higher risk of becoming overweight (81).

Furthermore, in the current study a lower consumption of dried fruits was associated with higher BMI_pct in males. It was reported that males eat less fruits than females and in this survey the preferences for fruit and vegetable was the main predictor (81%) of the sex difference here (16). Generally, independent of sex, a lower intake of fruits and vegetables which are high in fibre (and also vitamins and minerals) is associated with overweight in children (19) and also in adults (86, 87). In the survey on children‘s eating behavior (*n* = 39), it was shown that children with overweight and obesity ate significantly less fruits and vegetables than children with normal weight, but no sex differences for intake were found here (19). However, in the survey of Fogel and Blisserr (*n* = 99), the intake of fruits and vegetables did not show a significant correlation with BMI in 5–9 year old school children and again did not report on sex differences (88).

Legumes are fibre-rich and we hypothesised that the consumption would be lower in children with higher BMI_pct, as fibres are known to facilitate earlier satiation and improve diet quality (89). Moreover, intake of legumes in adults is associated with other health benefits such as reduced risk of cancer, cardiovascular disease (90) and metabolic syndrome (11). In contrast, we found that the legume consumption, especially in females, was positively associated with BMI (Table 2) and was further included within GsLM (section Relationship between BMI-for-age percentiles and all other factors) models. This result was mainly influenced by data from UK and Spain and we assume the result is related to typical preparation methods and combination with energy dense foods and ingredients. In Spain, the traditional way to prepare legume-dishes involves adding stewed vegetables and pork (chorizo, blood sausage, and ribs). Particularly, the combination with meat might increase the energy density in these dishes. In the UK, this higher legume intake may have been partly driven by a higher consumption of pre-prepared soft baked beans and/or cooked lentils as Dhal, very popular, easy and fast cooked affordable food (91, 92). Baked beans are generally high in sugar (91, 92) and Dhal high in fat. If used frequently in the diet in place of non-sugared/low-fat vegetables and legumes, it is perhaps more likely to contribute to higher rather than lower weight status. Moreover, our recent finding that children with higher legume consumption like softer texture fits here—as canned beans and boiled legumes are soft in their texture (40).

### Limitations

A few limitations could have affected our results such as that we mainly analysed BMI from self-reported data. Here, to consider a qualitative procedure child‘s weight and height was asked via the same question in all countries. Therefore, correlation of self-reported BMI-data vs. measured ones could only be analysed for Austria and UK. We want to acknowledge that consistency in measurement is fundamental in clinical and epidemiological research and that discrepancy and bias might have effect our BMI data. Furthermore, different classification methods limit the comparability with other study results on BMI. The estimation of daily food intake in children is important and often performed by using a FFQ. This is a widely applied and relevant instrument in epidemiological investigations in assessing children nutrition (93). FFQs are simple to administer and cost-efficient, although have limited validity and reliability in the context of over- and underestimation and overly rely on subject‘s memory (94). Some of these limitations are exacerbated where the questionnaire incorporates a higher number of food items. The use of indices which summarise food items and groups are of interest to reduce complexity and have been shown to be valid in young children, as have FFQs which use shorter recall reporting periods (95). Furthermore, using pictures combined with portion sizes, clear instructions and question period (e.g., last month) are relevant in valid food questionnaire instruments, especially in epidemiological approaches (96, 97). Therefore, in this study we utilised a FFQ that was short be containing only 17 items of direct relevance to the study, it asked parents only to remember the last 4 week period, and it provided images of each food item. The item number might have been too few to determine more specific differences (e.g., for legumes) and information on preparation methods would have been helpful. A further improvement in future studies would be to add portion size. As exact fibre intake could not be calculated, we were only able to estimate the intake of foods by daily frequencies portions. Very few children in the study consumed a high proportion of wholegrain foods. Where significant relationships were found between consumption of wholemeal products and BMI_pct, it must be noted that the consumption data contained outliers. Whereas, half of the children in the study consumed less than half of a daily equivalent of wholemeal alternatives (wholegrain bread, cereals, biscuits, rice or pasta), there were only 23 children (7%) reported to consume 2 or more DFEs of these alternatives. Once the outlying data were removed, secondary analysis (GsLM) found no significant relationship between wholegrain consumption and BMI-pct. We suggest this justifies the need for intervention studies of children in this age group comparing high wholegrain diets to standard diets. Furthermore, as food high in fibre also contains minerals, vitamins, and phytochemicals which are known to have positive health effects, this might additionally influence to the BMI related outcomes. Finally, even though the sample size was appropriate overall (n=330), we acknowledge the fact that for some variables (e.g., country-related differences) a larger sample size would have been beneficial. Future studies should confirm and extend our findings, here, the parental compliance should be considered as we were faced with low response.

## Conclusions

Significant associations between parent-reported intake of fibre rich and also fibre poor foods and the child BMI-for-age percentile were found, but were depended on specific foods and sex. Overall there was a weak, but significant, negative association between the total consumption of wholemeal products (wholegrain equivalent of bread, cereals, biscuits, rice, and pasta) and weight status. Furthermore, there was no direct evidence that texture preference for soft and hard food is associated with BMI_pct of 9–12-year-old children. Moreover, cultural differences and sex need to be considered as determinants of food preferences. Further research encompassing a wider range of single foods including preparation methods and full recipes could assist in gaining deeper insights in the relationship of weight status, fibre consumption, and texture preferences of children.

## Data Availability Statement

The raw data supporting the conclusions of this article may be made available by the authors pending a specific request at the discretion of the Data Access and Management Commission of the E3S - Working group on Children.

## Ethics Statement

The studies involving human participants were reviewed and approved by Austria: No. 30-200 ex. 17/18, Finland: No. 12/2018, Italy: No. 49/17, Spain: No. PI2017180, Sweden: No 2017/549, UK: No. UREC 18/15. Written informed consent to participate in this study was provided by the participants' legal guardian/next of kin.

## Author Contributions

HJ, ML, VA, MS, PS, and GZ: conceptualisation. RK, BA, ML, MS, PS, GZ, and LM: data curation. MH-W, BA, ML, VA, MS, and PS: funding acquisition. MH-W, RK, and LM: writing–original draft. BA, HJ, ML, VA, MS, PS, and GZ: writing–review & editing. All authors contributed to the article and approved the submitted version.

## Conflict of Interest

The authors declare that the research was conducted in the absence of any commercial or financial relationships that could be construed as a potential conflict of interest.
